# Glutathione Transferase P1: Potential Therapeutic Target in Ovarian Cancer

**DOI:** 10.3390/medicina58111660

**Published:** 2022-11-16

**Authors:** Petar Simic, Igor Pljesa, Lazar Nejkovic, Djurdja Jerotic, Vesna Coric, Jelena Stulic, Nenad Kokosar, Dunja Popov, Ana Savic-Radojevic, Vladimir Pazin, Marija Pljesa-Ercegovac

**Affiliations:** 1 Obstetrics and Gynaecology Clinic Narodni Front, 11000 Belgrade, Serbia; 2 Gynaecology and Obstetrics Centre Dr Dragiša Mišović, 11000 Belgrade, Serbia; 3 Faculty of Medicine, University of Belgrade, 11000 Belgrade, Serbia; 4 Institute of Medical and Clinical Biochemistry, 11000 Belgrade, Serbia

**Keywords:** ovarian cancer, chemoresistance, glutathione S transferase P1, microRNA, apoptosis

## Abstract

Chemotherapy resistance of ovarian cancer, regarded as the most lethal malignant gynecological disease, can be explained by several mechanisms, including increased activity of efflux transporters leading to decreased intracellular drug accumulation, increased efflux of the therapeutic agents from the cell by multidrug-resistance-associated protein (MRP1), enhanced DNA repair, altered apoptotic pathways, silencing of a number of genes, as well as drug inactivation, especially by glutathione transferase P1 (GSTP1). Indeed, GSTP1 has been recognized as the major enzyme responsible for the conversion of drugs most commonly used to treat metastatic ovarian cancer into less effective forms. Furthermore, GSTP1 may even be responsible for chemoresistance of non-GST substrate drugs by mechanisms such as interaction with efflux transporters or different signaling molecules involved in regulation of apoptosis. Recently, microRNAs (miRNAs) have been identified as important gene regulators in ovarian cancer, which are able to target GST-mediated drug metabolism in order to regulate drug resistance. So far, miR-186 and miR-133b have been associated with reduced ovarian cancer drug resistance by silencing the expression of the drug-resistance-related proteins, GSTP1 and MDR1. Unfortunately, sometimes miRNAs might even enhance the drug resistance in ovarian cancer, as shown for miR-130b. Therefore, chemoresistance in ovarian cancer treatment represents a very complex process, but strategies that influence GSTP1 expression in ovarian cancer as a therapeutic target, as well as miRNAs affecting GSTP1 expression, seem to represent promising predictors of chemotherapeutic response in ovarian cancer, while at the same time represent potential targets to overcome chemoresistance in the future.

## 1. Introduction

Ovarian cancer (OC) is regarded as the most lethal malignant gynecological disease, with overall survival in the range of 45–50% [1,2]. Ranked as the second most common gynecological cancer, ovarian cancer is heterogeneous in nature and divided into three major histopathological groups, of which the epithelial subgroup comprises approximately 90% of the cases worldwide [3]. According to statistics, it is estimated that annually worldwide, 230,000 women will be diagnosed with this disease, with lethal outcomes in approximately 150,000 [4]. A great deal of research has been performed towards elucidating the malignant and silent nature of ovarian cancer, and both genetic and epigenetic factors are shown to influence the progression of the disease. Indeed, approximately 10–15% of familial OCs are consequential to BRCA1 and BRCA2 gene mutations [5], while the presence of mutation in TP53 tumor-suppressor gene is found in 60–80% of both familial and sporadic OC cases [6]. The leading cause of survival rates below 50% are advanced-stage disease at the time of diagnosis, chemoresistance, and lack of centralized care for patients, especially in developing counties [7,8]. Regarding the time of diagnosis, detection in the early stage is not satisfactory due to the fact that the most-used diagnostic tools for OC screening, such as transvaginal ultrasound and blood test for CA125 tumor marker, are not efficient enough [9]. Moreover, the major challenges associated with the development of a clinically applicable screening strategy are, on one side, the low prevalence of ovarian cancer and, on the other side, the lack of biomarkers with appropriate sensitivity and specificity. When it comes to treatment, fundamental principles are based on radical surgery combined with platinum-based chemotherapy (carboplatin or cisplatin) in combination with taxane (paclitaxel and docetaxel), to which most patients are initially responsive but, due to development of platinum resistance, a relapse occurs in approximately 80% of OC patients [10]. In platinum-based chemotherapy-resistant ovarian carcinoma, the treatment is based on usage of gemcitabine, doxorubicin, and bevacizumab [3]. Overall, primary debulking surgery still represents a cornerstone therapy, whereas the tumor burden after the surgery is considered the most important survival factor [11,12].

In recent years, great strides have been made in clarifying the molecular background of ovarian cancer, as well as for understanding the mechanism of drug resistance in OC patients. Well-conduced clinical trials have paved the way for the introduction of novel target therapy, primarily antiangiogenic agents, followed by inhibitors against poly (ADP-ribose) polymerase (PARP) molecules involved in the DNA damage repair processes [13,14]. Although the advanced level treatment options include targeted therapy, immunotherapy, and hormone therapy, chemotherapy is still considered the most vital part of treatment in metastatic ovarian cancer [15].

## 2. Chemotherapy Resistance of Ovarian Cancer

Regarded as a complex phenomenon, which leads to the development of tolerance and failure in cellular response to treatment with one or multiple chemotherapeutic agents, drug resistance or chemotherapy resistance represents a great concern in everyday clinical practice [16]. Moreover, apart from developing resistance to the applied chemotherapeutic agent, cancer cells may even develop simultaneous cross-resistance to a wide range of drugs that may even be functionally and structurally unrelated to the applied chemotherapeutics [17].

**Figure 1:**
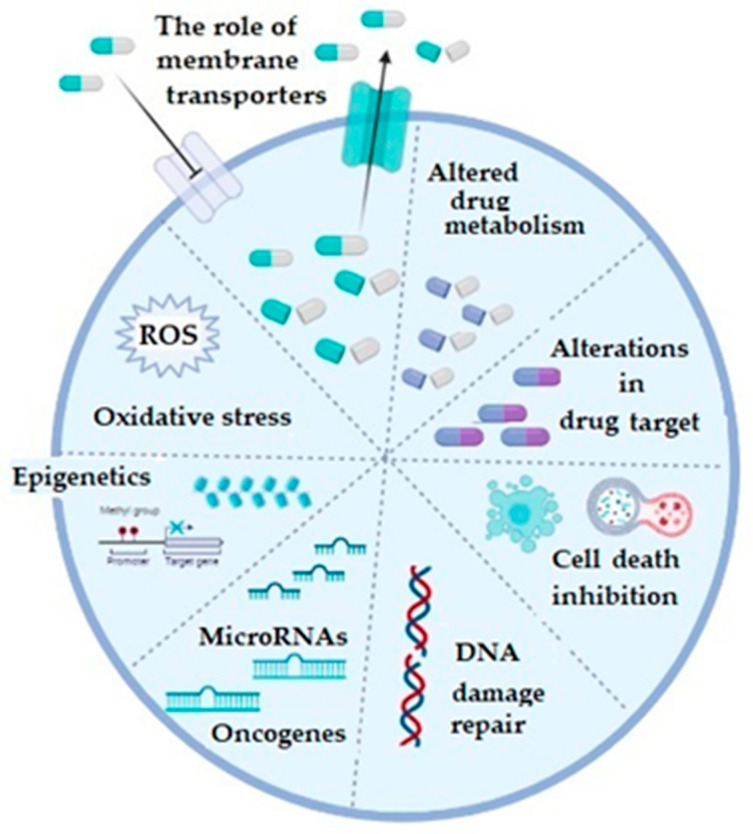
The illustrative summary of cellular mechanisms which are implicated in drug resistance in ovarian cancer; ROS: reactive oxygen species; figure created with Biorender (https://biorender.com/, accessed on 6 November 2022).

In general, mechanisms of chemoresistance are stratified into two basic categories, including de novo or intrinsic and acquired or extrinsic chemoresistance [16,18]. As in any other cancer, chemotherapy resistance of ovarian cancer can be explained by several basic mechanisms, including increased activity of efflux transporters, leading to decreased intracellular drug accumulation, increased efflux of the therapeutic agents from the cell by multidrug-resistance-associated protein (MRP1) [19], enhanced DNA repair, altered apoptotic pathways, silencing of a number of genes, as well as increased cellular levels of glutathione (GSH) and glutathione transferases (GSTs), which are involved in drug detoxification processes (platinum agents and taxol) and seem to play a very important role in this phenomenon [3,16,20,21] (Figure 1).

Regarding the membrane transporters, both influx and efflux, they participate in the chemoresistance mechanism, which is considered as the most prevalent one and which is based on the reduced cellular accumulation of the applied chemotherapeutic [16,22]. The majority of efflux transporters considered responsible for transporting the drugs outside the ovarian cancer cells, such as doxorubicin, vincristine, cisplatin, paclitaxel, topotecan, and etopiside, belong to a protein superfamily of ATP-binding cassette or ABC transporters [3,23]. Multidrug resistance (MDR)-associated proteins (MRPs), especially the MRP1 (encoded by ABCC1) and MRP2 (encoded by ABCC2) genes, as well as the ATP-dependent glycoprotein P-gp (encoded by ABCB1 gene) and breast cancer resistance protein BCRP (encoded by ABCG2 gene) gained most attention in OC [3]. Increased expression of any of these efflux transporters decreases intracellular concentration of the corresponding drug in OC. Influx or uptake transporters, on the other hand, belong to a wide group of solute carriers (SLC) transporter families, among which organic-anion-transporting proteins (OATPs), especially OATP1B3, is considered important in OC. Precisely, its expression is associated with influx of cis-, cabo-, and oxaliplatin [24]. Additionally, folate receptor α and the reduced folate carrier (RFC) may also be important as differential regulators for the development and progression of ovarian cancer. Namely, in epithelial OC, folate receptor α is highly expressed and increases with the stage of the disease [25,26,27].

Regarding the detoxification pathways of platinum derivatives as well as paclitaxel, as the drugs most commonly used to treat metastatic ovarian cancer patients, glutathione transferases (GSTs) have been recognized as the major enzymes responsible for the conversion of these drugs into less effective forms [28]. Glutathione transferases are a large family of enzymes responsible for catalyzing the conjugation of xenobiotics, including anticancer drugs, with glutathione [29,30]. Great inter-individual differences exist in the GST isoenzyme profile, due to the fact that almost all members of cytosolic GSTs exhibit genetic polymorphism [29,30]. As a consequence, complete lack or alteration in GST enzyme activity might affect the capacity for biotransformation in certain individuals, making them more prone to cancer development. Single nucleotide polymorphisms (SNPs) are mostly responsible for variations identified within genes encoding for cytosolic GSTs and, furthermore, they were associated with numerous diseases, including cancer [29]. In the case of GST pi (GSTP1), SNP leading to amino acid substitution from isoleucine (Ile) to valine (Val) changes catalytic and regulatory properties of the GSTP1 enzyme. Regarding alpha class GST (GSTA1), polymorphism is represented by three, apparently linked, SNPs: −567TOG, −69COT, and −52GOA, which lead to differential expression with lower transcriptional activation of the variant GSTA1*B (−567G, −69T, and −52A) than the common GSTA1*A alleles (−567T, −69C, and −52G). One more SNP, precisely the substitution of Ala to Asp at position 140, changes the deglutathionylase and thioltransferase activity of GST omega class (GSTO1), while, similarly to GSTA1, single nucletide polymorhism (A to G), leading to Asn to Asp substitution at position 142, is related to altered protein levels of GSTO2. On the other hand, deletion polymorphisms of genes encoding for human cytosolic GSTM1 and GSTT1 are rather common in human populations. Approximately half of the population lacks GSTM1 enzyme activity, due to a homozygous deletion of the GSTM1 gene, while in the case of GSTT1, gene homozygous deletion, with consequential lack of GSTT1 enzyme activity, is present in approximately 20% of Caucasians (Table 1) [29,31,32,33,34].

Apart from platinum derivatives, GSTs are involved in the development of chemoresistance by detoxification of numerous other chemotherapeutics [31,35], including chlorambucil, cyclophosphamide, melphalan, thiotepa, etc., which are recognized as substrates for GSTs and can be directly inactivated through GST-dependent conjugation reactions (Table 2) [31,36].

**Figure 2:**
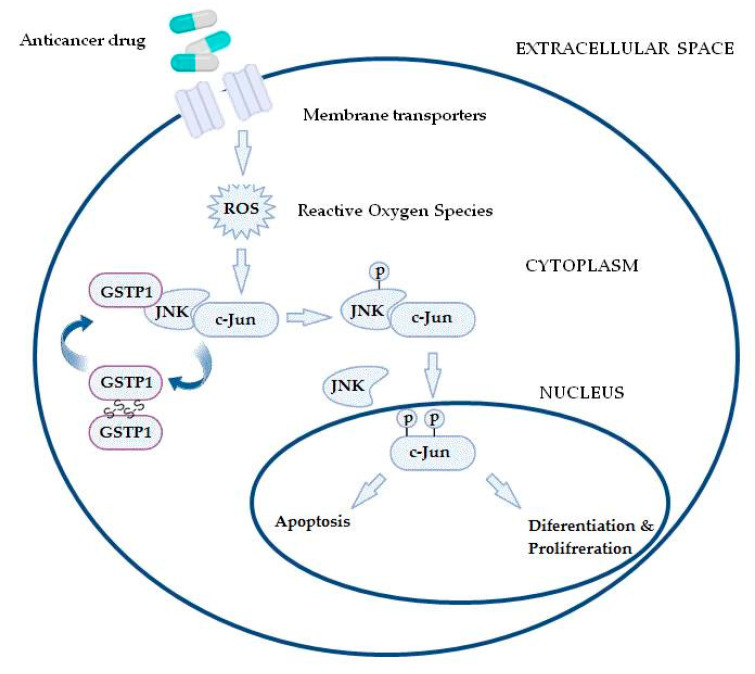
In the case of increased reactive oxygen species (ROS) content, the GSTP1:JNK1 complex dissociates and leads to GSTP1 oligomerisation. Consequently, activated JNK1 causes c-Jun phosphorylation, resulting in its nucleus translocation and initiation of alternative processes, depending on the level of ROS.

Furthermore, GSTs may even be responsible for chemoresistance of non-GST substrate drugs by mechanisms such as interaction with efflux transporters or different signaling molecules involved in regulation of apoptosis [31,37,38,39]. This especially refers to pi class GST (GSTP1), since GSTP1 possesses binding activity toward small and macromolecules, acts as a negative regulator of kinase-dependent apoptotic signaling pathways by forming protein–protein complexes with regulatory mitogen-activated kinases such as JNK1 (c-Jun NH2-terminal kinase), and, in addition to its role in detoxification of potential cancerogenic substances, GSTP1 is capable of increasing drug efflux from the cell, thus contributing to chemoresistance (Figure 2) [30,31,40]. Namely, through interaction with MRP-1, GSTP1 exhibits a synergistic effect on chemoresistance development to ethacrynic acid, chlorambucil, vincristine, and etoposide [41]. Other classes of GSTs, primarily GSTA1 and GSTM1, can contribute to chemoresistance mechanisms as well. Due to the structural homology between GSTA1 and GSTP1, GSTA1 may also suppress JNK1 signaling, while this class of GSTs also contributes to chlorambucil chemoresistance [42]. Similarly, through synergism of GSTM1 and MRP-1, cancer cells are protected from vincristine effects [43]. Furthermore, GSTM1 is capable of forming protein–protein interactions with either apoptosis signal—regulating kinase (ASK1) or thioredoxin (Trx)—in that way contributing to cellular redox-sensitive dynamic equilibrium [31]. Interestingly, by forming protein–protein interaction with tumor necrosis factor receptor-associated factor 2 (TRAF2), GSTP1 prevents ASK1:TRAF2 interaction and, consequently, ASK1 activation. Taken together, it might be proposed that overexpressed glutathione transferases via their catalytic, regulatory, and/or synergistic roles participate in several major mechanisms of chemoresistance.

## 3. GSTP1 Expression in Ovarian Cancer

Since ovarian cancer is thought to result from an accumulation of genetic changes [44], identification of inter-individual genetic variations, especially in genes encoding enzymes involved in inactivation of genotoxic substances, gained much attention. Namely, it is believed that this might enable early detection of the disease, significant changes in long-term survival, as well as personalized individual treatment in patients with ovarian cancer [45]. For that reason, the cytosolic classes M1, T1, and especially P1 gained most attention in ovarian cancer [28,46,47].

As mentioned, GSTP1 single nucleotide polymorphism (SNP) rs1695, results in amino acid substitution from isoleucine (*Ile*) to valine (*Val*) [48] and can affect both its catalytic and non-catalytic activity [31]. Although the carriers of the GSTP1*Ile105 allele have a higher catalytic efficiency for standard GST substrate (1-chloro-2,4-dinitrobenzene) than the carriers of *Val105 variant [49], the latter seems to confer higher catalytic efficiency in detoxification of polycyclic aromatic hydrocarbon (PAH) diol epoxide, present in tobacco smoke [50]. Since, GSTP1 also participates in the regulation of stress signaling and apoptosis via its non-catalytic activity [38], the substitution of amino acid isoleucine (*Ile*) with valine (*Val*) at position 105 can alter the GSTP1-mediated inhibitory effect of JNK activity. On the basis of the results on increased ovarian cancer risk in GSTP1*Ile (referent) allele carriers, it might be speculated that the stronger GSTP1:JNK interaction, which includes participation of GSTP1-risk associated allele, could prevent activation of apoptosis of ovarian cancer cells in these women, further affecting the progression of disease [51]. Furthermore, the data on 6-fold-increased ovarian cancer risk in women carriers of combined “risk” genotypes (GSTT1-active/GSTP1*Ile) suggest a high probability of their synergetic risk effect on carcinogenesis in these women [51].

It is important to note that GST polymorphisms can also affect the prognosis and the efficacy of chemotherapy in ovarian cancer patients. Namely, Khrunin et al. showed that GSTP1* Ile105Val polymorphism was strongly associated with progression-free survival in OC [52]. Precisely, homozygous carriers of Ile/Ile genotype had an increased progression-free survival compared with those with one or two *Val* alleles. Moreover, it has been shown that the GSTP1*B allele is also involved in the development of drug resistance and, as suggested in the study of Ghalia et al., high GSTP1 levels may be useful for monitoring during chemotherapy [53]. In this field, there are recent data on the beneficial effect of Hsp90 inhibitors in reversing cisplatin resistance of human ovarian cancer cell line (SKOV3), which was mediated by modifying the expression of multidrug-resistance-related genes, especially GSTP1, p53, Bcl-2, survivin, BRCA1, and BRCA2 [54,55].

Therefore, both catalytic and regulatory roles of GSTs might be regarded as important contributing factors in at least three major chemoresistance mechanisms. The fact that various cancer cells possess different and unique GST signature enables them to be suitable targets for the development of inhibitor drugs or prodrugs (Table 3) [31,36,54,56,57,58,59,60,61,62]. For that reason, the expression of GSTs at various levels has been studied in ovarian cancer for more than three decades. The data obtained have unambiguously shown that the level of GSTP1 especially is increased in ovarian cancerogenesis and related to chemoresistance of these tumors [46], which is demonstrated in both in vivo and in vitro settings. Indeed, development of cisplatin resistance in ovary adenocarcinoma (SKOV-3) is associated with significant increase in hGSTP1 expression [54,63]. Aiming to study the association between the GSTP1 and chemosensitivity of ovarian cancer, Sawers et al. introduced ovarian tumor cell line models [46]. They demonstrated that GSTP1 has an important role in cisplatin and carboplatin metabolism in ovarian cancer cells and that inter-tumor differences in GSTP1 expression directly influence response to platinum-based chemotherapy in ovarian cancer patients [64,65]. Therefore, stratification of ovarian cancer patients who might benefit from novel first-line therapies, depending on their detoxification capacity and the ability to simultaneously increase benefits and decrease toxicity of applied antitumor drugs, is of high importance. Namely, GSTP1 knockdown selectively influenced cisplatin and carboplatin chemosensitivity (2.3- and 4.83-fold change in IC50, respectively), and this effect was mediated by significant reduction in cell invasion and migration, while cell cycle progression was unaffected [46,66]. Furthermore, the same group identified several novel GSTP1 target genes and candidate platinum chemotherapy response biomarkers [46].

The expression of GSTP1 in ovarian cancer tissue is studied mostly in association with other resistance proteins, especially MRP1 or lung-related protein [57]. In their recent well-designed study which enrolled 121 ovarian cancer patients, Tong et al. demonstrated that the expression levels of GSTP1 was lower in the chemotherapy-sensitive group than in the chemotherapy-resistant group of patients. Moreover, patients with high expression of GSTP1, MDRP1, and GSK3β mRNA had a much lower 3-year survival rate than patients with low expression of these genes, suggesting its importance as a prognostic factor [57]. Another clinical study aimed to investigate the role of GSTP1 in primary epithelial ovarian cancer. Conducted multivariate logistic regression indicated that the expression level of lung resistance protein (LRP) and GSTP1 genes was a risk factor for primary epithelial ovarian cancer prognosis. Furthermore, the expression of LRP and GSTP1 in the negative-group survival curves was higher compared with the positive group [19].

## 4. Strategies That Influence GSTP1 Expression in Ovarian Cancer as Therapeutic Target

Glutathione transferase P1 is considered a promising therapeutic target in ovarian cancer based on application of various strategies that affect either its expression or its detoxifying and signaling roles [31]. Thus, it has been shown that suppressing glucose-6-phosphate dehydrogenase (G6PD) using shRNA or an inhibitor, either as single agents or in combination, sensitized paclitaxel-resistant cancer cells to paclitaxel treatment and thereby improved its therapeutic efficacy via regulation of the GSTP1 expression [28]. Additionally, it seems that increased expression of GSTP1 in ovarian cancer is associated with non-coding RNA LINC00152 (LINC00152). Silencing of LINC00152 increased the apoptotic rates and enhanced the chemosensitivity of CoC1 and CoC1/DDP cells to cisplatin. Since LINC00152 silencing decreased the expression of MDRP1 as well as GSTP1, it is proposed as a potential novel therapeutic target related to downregulation of GSTP1 expression and ovarian cancer chemosensitising [67].

Another way to modulate GST expression and activity has recently been suggested by Sirota et al. [68]. Namely, caffeic acid, a non-toxic polyphenol which is abundant in many foods, seems to modulate GST and glutathione reductase (GSR) activity, both involved in resistance of cancer cells towards cisplatin [68]. Caffeic acid induces the nuclear factor (erythroid-derived 2)-like2 (Nrf2) pathway and can also inhibit the activity of GST and GSR. Importantly, GSTP1 is among Nrf2 target genes, while GSTP1 has a potential to form a GSTP1/Nrf2 protein complex, affecting Nrf2 stabilization and its further actions [40]. Sirota et al. demonstrated that the co-treatment of cancer cells with cisplatin and caffeic acid can enhance cisplatin cytotoxicity and increases the amount of platinum bound to nuclear DNA, while 6 h of pre-incubation with caffeic acid prior to cisplatin treatment led to acquired resistance to cisplatin and reduced DNA binding. These results suggested that the enzyme inhibitory action of caffeic acid is dominant when the two agents are co-administered leading to increased cytotoxicity, and the Nrf2 induction is dominant when the cells are treated with caffeic acid prior to cisplatin treatment leading to resistance [68].

**Figure 3:**
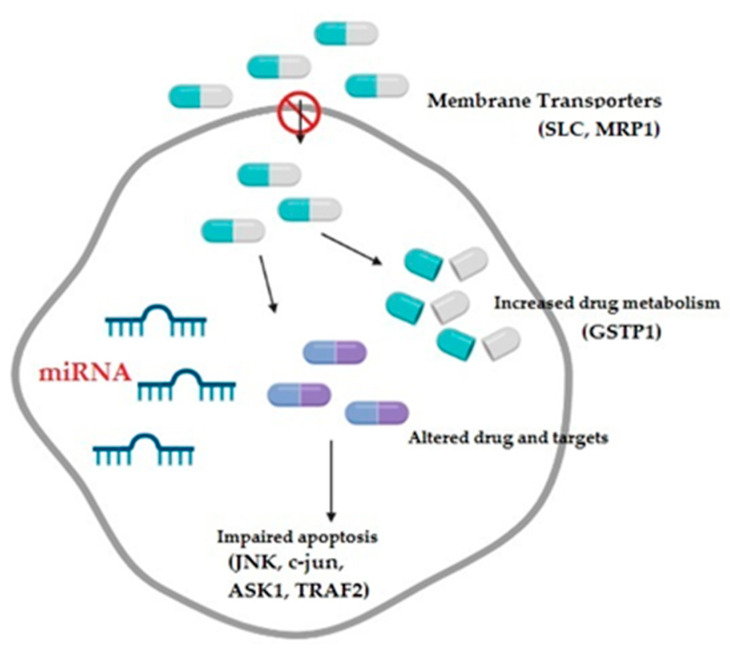
The mechanisms contributing to the development of multidrug resistance in ovarian cancer comprise decreased drug intake, increased drug metabolism, altered drug targets, and impaired apoptotic pathways, all potentially modulated by microRNAs; SLC: solute carriers transporter family; MRP1: multidrug-resistance-associated protein; GSTP1: glutathione transferase P1; JNK: c-Jun NH2-terminal kinase; ASK1: apoptosis signal-regulating kinase; and TRAF2: tumor necrosis factor receptor-associated factor 2.

Small non-coding RNAs or microRNAs (miRNAs) have also recently been identified as a novel class of gene regulators, playing an important role in various malignancies including ovarian cancer [69] (Figure 3). Since they participate in various biological processes, as well as post-transcriptional gene regulation, it has been shown that their dysregulation either via genetic or epigenetic modifications might contribute to cancer development [70,71]. Available data suggest that they might be regarded either as oncogenes or tumor-suppressor genes, depending on their specific role (e.g., cell survival, apoptosis, cell senescence, DNA damage repair, or p53-related network) and level of expression (upregulated or downregulated), and they also participate in chemoresistance development [16,72,73].

Numerous miRNAs have been investigated due to their potential involvement in ovarian cancer chemoresistance, including micro-RNA 9, miRNA-21, miRNA-21-3p, miRNA-27a, miRNA-29, miRNA-30a, miRNA-31, miRNA-93, miRNA-125a, miRNA-130a, miRNA-133b, miRNA-136, miRNA-145, miRNA-149, miRNA-150, miRNA-182, miRNA-185, miRNA-186, miRNA-200 family, miRNA-214, miRNA-376c, miRNA-513a-3p, and many others [16,72,73,74].

Among microRNAs investigated in ovarian cancer, which are shown to regulate GSTP1 gene expression, is miR-186. Data indicate that mRNA and protein expression levels of MDR1 and GSTP1 were downregulated after transfection with miR-186, while upregulated following anti-miR-186 transfection, which demonstrates that this miRNA might sensitize ovarian cancer cells to paclitaxel and cisplatin by targeting both MDR1 and modulating the expression of GSTP1 [75].

MicroRNA-133b also targets GSTP1 expression to increase ovarian cancer cell sensitivity to chemotherapy drugs. Namely, the expression of miR-133b was significantly lower in primary resistant ovarian carcinomas than in the chemotherapy-sensitive carcinomas, which was also confirmed in primary resistant ovarian cell lines (A2780/Taxol and A2780/DDP) [76]. However, after miR-133b transfection, cell lines showed increased sensitivity to paclitaxel and cisplatin, while anti-miR-133b transfection reduced cell sensitivity to paclitaxel and cisplatin. Interestingly, dual-luciferase reporter assay showed that miR-133b interacted with the 3’-untranslated region of GSTP1, which explains why mRNA and protein levels of MDR1 and GSTP1 were downregulated after miR-133b transfection and, vice versa, upregulated after anti-miR-133b transfection. This led to the conclusion that this is one more microRNA that might reduce ovarian cancer drug resistance by silencing the expression of the drug-resistance-related proteins, GSTP1 and MDR1 [69,76].

These data are in line with several studies in other types of cancer, which have shown that miRNAs are able to target GST-mediated drug metabolism in order to regulate drug resistance. Indeed, in lung carcinoma cells A549, Zhang et al. reported that miRNA-513a-3p could negatively regulate GSTP1 gene expression, suggesting that overexpression of miR-513a-3p resensitized cisplatin-resistant cancer cells [77].

In contrast to these studies which promote miRNAs as chemosensitising agents, it seems that sometimes they may even enhance the drug resistance, as in case of miR-130b. Namely, when human ovarian carcinoma cell line A2780 and paclitaxel-resistant A2780/Taxol cells were exposed to cisplatin or paclitaxel in the presence or absence of transfected miR-130b, higher expression levels of miR-130b were found in A2780/Taxol cells than in A2780 cells, which surprisingly decreased sensitivity to paclitaxel and cisplatin compared with mock-transfected and negative control cancer cells. However, mRNA expression levels of MDR1 and GSTP1 and the protein expression levels of MDR1 and GSTP1 were downregulated following miR-130b transfection, which still suggested that miRNA-130b may be involved in the development of drug resistance in ovarian cancer cells [78].

Taken together, chemoresistance in ovarian cancer treatment is a very complex multi-factorial process which includes many different underlying mechanisms. Introduction of new technologies, as well as innovative targeted therapies, enables novel mechanisms to overcome development of chemoresistance in this cancer, which exhibits high level of molecular heterogeneity. Among promising candidates that might be helpful in the development of personalized therapies which are based on anticipation of cellular response to applied chemotherapeutics in ovarian cancer cells are miRNAs. Due to their extensive gene regulatory roles, it seems that they are able to regulate nearly all mechanisms underlying drug resistance in OC, including GSTP1 and MDR1. Therefore, apart from significant role of GSTP1 in drug metabolism and membrane transport, miRNAs affecting its expression could represent promising predictors of chemotherapeutic response in ovarian cancer, while at the same time, represent potential targets to overcome chemoresistance in the future. Importantly, apart from focusing on the chemoresistance phenomenon in standard therapeutic approaches based on platinum and paclitaxel, research interest should be directed toward understanding the resistance mechanisms of novel chemotherapeutics used in ovarian cancer treatment.

## Figures and Tables

**Table 1:** Distribution of common GST polymorphisms in humans.

Gene	rs	Genotype	Distribution in Caucasians (%)
GSTA1	rs3957357	GSTA1 AA GSTA1 AB/BB (low-activity) ^3^	38 62
GSTP1	rs1695	GSTP1 Ile105Ile GSTP1 Ile105Val/Val105Val (variant) ^4^	45 55
GSTO1	rs4925	GSTO1 Ala140Ala GST01 Ala140Asp/Asp140Asp(variant) ^4^	44 56
GSTO2	rs156697	GSTO2 Asn142Asn GSTO2 Asn142Asp/Asp142Asp(variant) ^4^	45 55
GSTT1	deletion	GST1 active ^1^ GSTT1 null ^2^	70-80 20-30
GSTM1	deletion	GSTM1 active GSTM1 null	50 50

^1^ Active, if at least one active allele present; ^2^ Null, if no active alleles present; ^3^ Low activity, if at least one *B* allele present; ^4^ and Variant, if at least one *Val/Asp/Asp* allele present.

**Table 2:** Substrate specificities of cytosolic GSTs regarding different chemotherapeutics.

GST Class	Alleles	Substrates
Alpha	GSTA1*A GSTA1*B GSTA2*A GSTA2*B GSTA2*C GSTA2*D GSTA2*E	melphalan, chlorambucil, thiotepa, BCNU, brostallicin, and busulphan
Mu	GSTM1*0 GSTM1*A GSTM1*B GSTM3*A GSTM3*B GSTM4*A GSTM4*B	brostallicin, BCNU, ethacrynic acid, and thiopurines
Pi	GSTP1*A GSTP1*B GSTP1*C GSTP1*D	cisplatin, brostallicin, chlorambucil, doxorubicin, ethacrynic acid, cyclophosphamide, and thiotepa
Theta	GSTT1*0 GSTT1*A GSTT1*B GSTT2*A GSTT2*B	BCNU

**Table 3:** Differential expression of GSTs in cancer.

Type of Cancer	GST Class			
	Alpha	Mu	Pi	Theta
Ovarian		decreased expression	increased expression	
Lung		increased expression	increased expression	
Colorectal	decreased expression	decreased expression	increased expression	
Urinary bladder		increased expression	increased expression	increased expression
Liver	decreased expression		increased expression	
Renal	decreased expression	increased expression	increased expression	
Prostate			decreased expression	
Glioma			increased expression	
Breast		increased expression	increased expression	

## Data Availability

Not applicable.

## References

[1] Lheureux S., Gourley C., Vergote I., Oza A.M. (2019). Epithelial ovarian cancer. Lancet.

[2] Siegel R.L., Miller K.D., Fuchs H.E., Jemal A. (2021). Cancer Statistics, 2021. CA Cancer J. Clin..

[3] Chandra A., Pius C., Nabeel M., Nair M., Vishwanatha J.K., Ahmad S., Basha R. (2019). Ovarian cancer: Current status and strategies for improving therapeutic outcomes. Cancer Med..

[4] Bray F., Ferlay J., Soerjomataram I., Siegel R.L., Torre L.A., Jemal A. (2018). Global cancer statistics 2018: GLOBOCAN estimates of incidence and mortality worldwide for 36 cancers in 185 countries. CA A Cancer J. Clin..

[5] Neff R.T., Senter L., Salani R. (2017). BRCA mutation in ovarian cancer: Testing, implications and treatment considerations. Ther. Adv. Med. Oncol..

[6] Zhang Y., Cao L., Nguyen D., Lu H. (2016). TP53 mutations in epithelial ovarian cancer. Transl. Cancer Res..

[7] Cliby W.A., Powell M.A., Al-Hammadi N., Chen L., Miller J.P., Roland P.Y., Mutch D.G., Bristow R.E. (2015). Ovarian cancer in the United States: Contemporary patterns of care associated with improved survival. Gynecol. Oncol..

[8] Kim K., Hernlund E., Hernadi Z., Révész J., Pete I., Szánthó A., Bodnar L., Madry R., Timorek–Lemieszczuk A., Bozanovic T. (2013). Treatment patterns, health care utilization, and costs of ovarian cancer in Central and Eastern Europe using a Delphi panel based on a retrospective chart review. Int. J. Gynecol. Cancer.

[9] Liberto J.M., Chen S.-Y., Shih I.-M., Wang T.-H., Wang T.-L., Pisanic T.R. (2022). Current and Emerging Methods for Ovarian Cancer Screening and Diagnostics: A Comprehensive Review. Cancers.

[10] Damia G., Broggini M. (2019). Platinum Resistance in Ovarian Cancer: Role of DNA Repair. Cancers.

[11] Du Bois A., Reuss A., Pujade-Lauraine E., Harter P., Ray-Coquard I., Pfisterer J. (2009). Role of surgical outcome as prognostic factor in advanced epithelial ovarian cancer: A combined exploratory analysis of 3 prospectively randomized phase 3 multicenter trials: By the Arbeitsgemeinschaft Gynaekologische Onkologie Studiengruppe Ovarialkarzinom (AGO-OVAR) and the Groupe d’Investigateurs Nationaux Pour les Etudes des Cancers de l’Ovaire (GINECO). Cancer.

[12] Kurnit K.C., Fleming G.F., Lengyel E. (2021). Updates and New Options in Advanced Epithelial Ovarian Cancer Treatment. Obstet. Gynecol..

[13] Haunschild C.E., Tewari K.S. (2020). Bevacizumab use in the frontline, maintenance and recurrent settings for ovarian cancer. Future Oncol..

[14] Nero C., Ciccarone F., Pietragalla A., Duranti S., Daniele G., Salutari V., Carbone M., Scambia G., Lorusso D. (2021). Ovarian Cancer Treatments Strategy: Focus on PARP Inhibitors and Immune Check Point Inhibitors. Cancers.

[15] Ortiz M., Wabel E., Mitchell K., Horibata S. (2022). Mechanisms of chemotherapy resistance in ovarian cancer. Cancer Drug Resist..

[16] Norouzi-Barough L., Sarookhani M.R., Sharifi M., Moghbelinejad S., Jangjoo S., Salehi R. (2018). Molecular mechanisms of drug resistance in ovarian cancer. J. Cell Physiol..

[17] Wang J., Seebacher N., Shi H., Kan Q., Duan Z. (2017). Novel strategies to prevent the development of multidrug resistance (MDR) in cancer. Oncotarget.

[18] David W., Chan M.X.L., Ngan H.Y.S. (2012). Mechanisms of Chemoresistance in Human Ovarian Cancer at a Glance. Gynecol. Obstet..

[19] Gao B., Yang F., Chen W., Li R., Hu X., Liang Y., Li D. (2019). Multidrug resistance affects the prognosis of primary epithelial ovarian cancer. Oncol. Lett..

[20] Cole S.P.C. (2014). Targeting Multidrug Resistance Protein 1 (MRP1, *ABCC1* ): Past, Present, and Future. Annu. Rev. Pharmacol. Toxicol..

[21] Robey R.W., Pluchino K.M., Hall M.D., Fojo A.T., Bates S.E., Gottesman M.M. (2018). Revisiting the role of ABC transporters in multidrug-resistant cancer. Nat. Rev. Cancer.

[22] Li Q., Shu Y. (2014). Role of solute carriers in response to anticancer drugs. Mol. Cell. Ther..

[23] Januchowski R., Sterzyńska K., Zaorska K., Sosińska P., Klejewski A., Brązert M., Nowicki M., Zabel M. (2016). Analysis of MDR genes expression and cross-resistance in eight drug resistant ovarian cancer cell lines. J. Ovarian Res..

[24] Lancaster C.S., Sprowl J.A., Walker A.L., Hu S., Gibson A.A., Sparreboom A. (2013). Modulation of OATP1B-type transporter function alters cellular uptake and disposition of platinum chemotherapeutics. Mol. Cancer Ther..

[25] Siu M.K.Y., Kong D.S.H., Chan H.Y., Wong E.S.Y., Ip P.P.-C., Jiang L., Ngan H.Y.S., Le X.-F., Cheung A.N.Y. (2012). Paradoxical Impact of Two Folate Receptors, FRα and RFC, in Ovarian Cancer: Effect on Cell Proliferation, Invasion and Clinical Outcome. PLoS ONE.

[26] Vergote I.B., Marth C., Coleman R.L. (2015). Role of the folate receptor in ovarian cancer treatment: Evidence, mechanism, and clinical implications. Cancer Metastasis Rev..

[27] Wallace-Povirk A., Hou Z., Nayeen J., Gangjee A., Matherly L.H. (2021). Folate Transport and One-Carbon Metabolism in Targeted Therapies of Epithelial Ovarian Cancer. Cancers.

[28] Feng Q., Li X., Sun W., Sun M., Li Z., Sheng H., Xie F., Zhang S., Shan C. (2020). Targeting G6PD reverses paclitaxel resistance in ovarian cancer by suppressing GSTP1. Biochem. Pharmacol..

[29] Hollman A.L., Tchounwou P.B., Huang H.-C. (2016). The Association between Gene-Environment Interactions and Diseases Involving the Human GST Superfamily with SNP Variants. Int. J. Environ. Res. Public Health.

[30] Board P.G., Menon D. (2013). Glutathione transferases, regulators of cellular metabolism and physiology. Biochim. Biophys. Acta (BBA)—Gen. Subj..

[31] Pljesa-Ercegovac M., Savic-Radojevic A., Matic M., Coric V., Djukic T., Radic T., Simic T. (2018). Glutathione Transferases: Potential Targets to Overcome Chemoresistance in Solid Tumors. Int. J. Mol. Sci..

[32] McIlwain C.C., Townsend D.M., Tew K.D. (2006). Glutathione S-transferase polymorphisms: Cancer incidence and therapy. Oncogene.

[33] Oakley A. (2011). Glutathione transferases: A structural perspective. Drug Metab. Rev..

[34] Board P., Coggan M., Johnston P., Ross V., Suzuki T., Webb G. (1990). Genetic heterogeneity of the human glutathione transferases: A complex of gene families. Pharmacol. Ther..

[35] Ambrosone C.B., Sweeney C., Coles B.F., Thompson A.P., McClure G.Y., Korourian S., Fares M.Y., Stone A., Kadlubar F.F., Hutchins L.F. (2001). Polymorphisms in glutathione S-transferases (GSTM1 and GSTT1) and survival after treatment for breast cancer. Cancer Res..

[36] Lo H.-W., Ali-Osman F. (2007). Genetic polymorphism and function of glutathione S-transferases in tumor drug resistance. Curr. Opin. Pharmacol..

[37] Laborde E. (2010). Glutathione transferases as mediators of signaling pathways involved in cell proliferation and cell death. Cell Death Differ..

[38] Tew K.D., Townsend D.M. (2012). Glutathione-s-transferases as determinants of cell survival and death. Antioxid. Redox Signal..

[39] Coric V.M., Simic T.P., Pekmezovic T.D., Basta-Jovanovic G.M., Savic-Radojevic A.R., Radojevic-Skodric S.M., Matic M.G., Suvakov S.R., Dragicevic D.P., Radic T.M. (2017). GSTM1 genotype is an independent prognostic factor in clear cell renal cell carcinoma. Urol. Oncol. Semin. Orig. Investig..

[40] Bartolini D., Galli F. (2016). The functional interactome of GSTP: A regulatory biomolecular network at the interface with the Nrf2 adaption response to oxidative stress. J. Chromatogr. B.

[41] O’Brien M., Kruh G.D., Tew K.D. (2000). The influence of coordinate overexpression of glutathione phase II detoxification gene products on drug resistance. J. Pharmacol. Exp. Ther..

[42] Smitherman P.K., Townsend A.J., Kute T.E., Morrow C.S. (2004). Role of multidrug resistance protein 2 (MRP2, ABCC2) in alkylating agent detoxification: MRP2 potentiates glutathione S-transferase A1-1-mediated resistance to chlorambucil cytotoxicity. J. Pharmacol. Exp. Ther..

[43] Depeille P., Cuq P., Mary S., Passagne I., Evrard A., Cupissol D., Vian L. (2004). Glutathione S-transferase M1 and multidrug resistance protein 1 act in synergy to protect melanoma cells from vincristine effects. Mol. Pharmacol..

[44] Lee J.-Y., Kim H.S., Suh D.H., Kim M.-K., Chung H.H., Song Y.-S. (2013). Ovarian cancer biomarker discovery based on genomic approaches. J. Cancer Prev..

[45] Bast R.C., Hennessy B., Mills G.B. (2009). The biology of ovarian cancer: New opportunities for translation. Nat. Rev. Cancer.

[46] Sawers L., Ferguson M.J., Ihrig B.R., Young H.C., Chakravarty P., Wolf C.R., Smith G.D.W. (2014). Glutathione S-transferase P1 (GSTP1) directly influences platinum drug chemosensitivity in ovarian tumour cell lines. Br. J. Cancer.

[47] Ferracini A.C., Lopes-Aguiar L., Lourenço G.J., Yoshida A., Lima C.S.P., Sarian L.O., Derchain S., Kroetz D.L., Mazzola P.G. (2021). GSTP1 and ABCB1 Polymorphisms Predicting Toxicities and Clinical Management on Carboplatin and Paclitaxel-Based Chemotherapy in Ovarian Cancer. Clin. Transl. Sci..

[48] Watson M.A., Stewart R.K., Smith G.B., Massey T.E., Bell D.A. (1998). Human glutathione S-transferase P1 polymorphisms: Relationship to lung tissue enzyme activity and population frequency distribution. Carcinogenesis.

[49] Hua X., O’Donnellb R., Srivastava S.K., Xiaa H., Zimniakc P., Nanduric B., Bleicher R.J., Awasthid S., Awasthi Y.C., Ji X. (1997). Active site architecture of polymorphic forms of human glutathione S-transferase P1-1 accounts for their enantioselectivity and disparate activity in the glutathione conjugation of 7beta,8alpha-dihydroxy-9alpha,10alpha-ox y-7,8,9,10-tetrahydrobenzo(a)pyrene. Biochem. Biophys. Res. Commun..

[50] Sundberg K., Dreij K., Seidel A., Jernström B. (2002). Glutathione conjugation and DNA adduct formation of dibenzo[a,l]pyrene and benzo[a]pyrene diol epoxides in V79 cells stably expressing different human glutathione transferases. Chem. Res. Toxicol..

[51] Pljesa I., Berisavac M., Simic T., Pekmezovic T., Coric V., Suvakov S., Stamatovic L., Matic M., Gutic B., Milenkovic S. (2017). Polymorphic expression of glutathione transferases A1, M1, P1 and T1 in epithelial ovarian cancer: A Serbian case-control study. J. BUON.

[52] Khrunin A.V., Moisseev A., Gorbunova V., Limborska S. (2010). Genetic polymorphisms and the efficacy and toxicity of cisplatin-based chemotherapy in ovarian cancer patients. Pharm. J..

[53] Ghalia A.A., Rabboh N.A., el Shalakani A., Seada L., Khalifa A. (2000). Estimation of glutathione S-transferase and its Pi isoenzyme in tumor tissues and sera of patients with ovarian cancer. Anticancer Res..

[54] Zhang Z., Xie Z., Sun G., Yang P., Li J., Yang H., Xiao S., Liu Y., Qiu H., Qin L. (2015). Reversing drug resistance of cisplatin by hsp90 inhibitors in human ovarian cancer cells. Int. J. Clin. Exp. Med..

[55] Fontana F., Carollo E., Melling G.E., Carter D.R.F. (2021). Extracellular Vesicles: Emerging Modulators of Cancer Drug Resistance. Cancers.

[56] Sau A., Pellizzari Tregno F., Valentino F., Federici G., Caccuri A.M. (2010). Glutathione transferases and development of new principles to overcome drug resistance. Arch. Biochem. Biophys..

[57] Tong X., Zhao J., Zhang Y., Mu P., Wang X. (2019). Expression levels of MRP1, GST-π, and GSK3β in ovarian cancer and the relationship with drug resistance and prognosis of patients. Oncol. Lett..

[58] Kiliç M., Ada A.O., Oğuztüzün S., Demirağ F., Çelik S., Biçakçioğlu P., Işcan M. (2017). Polymorphisms and Protein Expressions of Glutathione S-Transferase M1 and T1 in Non-Small Cell Lung Cancer. Turk. J. Pharm. Sci..

[59] Beyerle J., Frei E., Stiborova M., Habermann N., Ulrich C.M. (2015). Biotransformation of xenobiotics in the human colon and rectum and its association with colorectal cancer. Drug Metab. Rev..

[60] Wang F., Zhang C., Zhu X., Zhang D., Zhang Z., Ni S., Wang Z., Xu S., Lan X., Ding Y. (2022). Overexpression of GSTP1 promotes colorectal cancer cell proliferation, invasion and metastasis by upregulating STAT3. Adv. Clin. Exp. Med..

[61] Tong Y., Yu Y., Zheng H., Wang Y., Xie S., Chen C., Lu R., Guo L. (2021). Differentially Expressed Genes in Clear Cell Renal Cell Carcinoma as a Potential Marker for Prognostic and Immune Signatures. Front. Oncol..

[62] Tsyganov M.M., Ibragimova M.K., Garbukov E.Y., Tsydenova I.A., Gaptulbarova K.A., Dolgasheva D.S., Zdereva E.A., Frolova A.A., Slonimskaya E.M., Litviakov N.V. (2022). Predictive and Prognostic Significance of mRNA Expression and DNA Copies Aberrations of ERCC1, RRM1, TOP1, TOP2A, TUBB3, TYMS, and GSTP1 Genes in Patients with Breast Cancer. Diagnostics.

[63] Kalinina E.V., Berozov T.T., Shtil A.A., Chernov N.N., Glasunova V.A., Novichkova M.D., Nurmuradov N.K. (2012). Expression of genes of glutathione transferase isoforms GSTP1-1, GSTA4-4, and GSTK1-1 in tumor cells during the formation of drug resistance to cisplatin. Bull. Exp. Biol. Med..

[64] Van Zyl B., Tang D., Bowden N.A. (2018). Biomarkers of platinum resistance in ovarian cancer: What can we use to improve treatment. Endocr. Relat. Cancer.

[65] Baert T., Ferrero A., Sehouli J., O’Donnell D., González-Martín A., Joly F., van der Velden J., Blecharz P., Tan D., Querleu D. (2021). The systemic treatment of recurrent ovarian cancer revisited. Ann. Oncol..

[66] Ledermann J.A., Pujade-Lauraine E. (2019). Olaparib as maintenance treatment for patients with platinum-sensitive relapsed ovarian cancer. Ther. Adv. Med. Oncol..

[67] Zou H., Li H. (2019). Knockdown of long non-coding RNA LINC00152 increases cisplatin sensitivity in ovarian cancer cells. Exp. Ther. Med..

[68] Sirota R., Gibson D., Kohen R. (2017). The timing of caffeic acid treatment with cisplatin determines sensitization or resistance of ovarian carcinoma cell lines. Redox Biol..

[69] An X., Sarmiento C., Tan T., Zhu H. (2017). Regulation of multidrug resistance by microRNAs in anti-cancer therapy. Acta Pharm. Sin. B.

[70] Forterre A., Komuro H., Aminova S., Harada M. (2020). A Comprehensive Review of Cancer MicroRNA Therapeutic Delivery Strategies. Cancers.

[71] O’Brien J., Hayder H., Zayed Y., Peng C. (2018). Overview of MicroRNA Biogenesis, Mechanisms of Actions, and Circulation. Front. Endocrinol..

[72] Zhao H., Liu S., Wang G., Wu X., Ding Y., Guo G., Jiang J., Cui S. (2015). Expression of miR-136 is associated with the primary cisplatin resistance of human epithelial ovarian cancer. Oncol. Rep..

[73] Zhan Y., Xiang F., Wu R., Xu J., Ni Z., Jiang J., Kang X. (2015). MiRNA-149 modulates chemosensitivity of ovarian cancer A2780 cells to paclitaxel by targeting MyD88. J. Ovarian Res..

[74] Zhao H.-M., Wei W., Sun Y.-H., Gao J.-H., Wang Q., Zheng J.-H. (2015). MicroRNA-9 promotes tumorigenesis and mediates sensitivity to cisplatin in primary epithelial ovarian cancer cells. Tumour Biol..

[75] Sun K.-X., Jiao J.-W., Chen S., Liu B.-L., Zhao Y. (2015). MicroRNA-186 induces sensitivity of ovarian cancer cells to paclitaxel and cisplatin by targeting ABCB1. J. Ovarian Res..

[76] Chen S., Jiao J.-W., Sun K.-X., Zong Z.-H., Zhao Y. (2015). MicroRNA-133b targets glutathione S-transferase π expression to increase ovarian cancer cell sensitivity to chemotherapy drugs. Drug Des. Dev. Ther..

[77] Zhang X., Zhu J., Xing R., Tie Y., Fu H., Zheng X., Yu B. (2012). miR-513a-3p sensitizes human lung adenocarcinoma cells to chemotherapy by targeting GSTP1. Lung Cancer.

[78] Zong C., Wang J., Shi T.-M. (2014). MicroRNA 130b enhances drug resistance in human ovarian cancer cells. Tumour Biol..

